# Are river protected areas sufficient for fish conservation? Implications from large-scale hydroacoustic surveys in the middle reach of the Yangtze River

**DOI:** 10.1186/s12898-019-0258-4

**Published:** 2019-09-25

**Authors:** Xiao Xie, Hui Zhang, Chengyou Wang, Jinming Wu, Qiwei Wei, Hao Du, Junyi Li, Huan Ye

**Affiliations:** 1 College of Fisheries, Huazhong Agricultural University, Wuhan, 430070 Hubei China; 2 Key Laboratory of Freshwater Biodiversity Conservation, Ministry of Agriculture of China, Yangtze River Fisheries Research Institute, Chinese Academy of Fishery Sciences, Wuhan, 430223 Hubei China

**Keywords:** Systematic conservation planning, Marxan, Hydroacoustic, Freshwater protected area, Reserve

## Abstract

**Background:**

The Yangtze River is the third largest river in the world and suffers from extensive anthropogenic impacts. The fishes in the Yangtze River are essential for the sustainable development of freshwater fisheries and the conservation of aquatic biodiversity in China. However, the fishery resources in the Yangtze River Basin have shown rapid decline due to various human activities. In recent years, nature reserves and germplasm resource reserves have become important means to protect fishes in the Yangtze River. However, nature reserves and germplasm resource reserves that regard freshwater fishes as the main object of protection are not common and have been rarely studied in China. In this paper, a hydroacoustic method and systematic conservation planning tool (Marxan) were combined to evaluate the effectiveness of reserves based on the spatial and temporal patterns of mature fishes in the middle reach of the Yangtze River (MRYR) from 2010 to 2017.

**Results:**

The hydroacoustic survey results indicated that in the longitudinal direction, low densities of mature fish species were observed in the Jingzhou (S2) and Jianli (S4, S5, S6) sections, whereas high densities of fish were observed in other sections, such as the Yichang (S1), Chenglingji to Huangsangkou (S7–S12), and Hukou (S15) sections. Among the regions preferred by fish, S7, S10 and S12 were non-reserves. No significant difference in mature fish density was observed between the non-reserves and nature reserves, and a similar result was obtained between the non-reserves and germplasm resource reserves. In Marxan, the optimal conservation sites selected for habitat restoration, such as the Chenglingji, Dengjiakou, Zhuankou, Hankou, Yangluo, and Huangsangkou sections, which are located in non-reserves, were identified in the MRYR.

**Conclusions:**

The Chenglingji, Dengjiakou, Zhuankou, Hankou, Yangluo, and Huangsangkou sections, which are located in non-reserves, play equally important roles in the conservation of fish populations in the MRYR. Our results indicated that further optimization is urgently needed for the currently protected areas in this region. These areas should be designated as reserves, and classification protection mechanisms should be adopted to strengthen the effectiveness of fish conservation in the MRYR.

## Background

Globally, freshwater ecosystems face multiple anthropogenic stressors. As a result, the rich biological resources in these ecosystems are dramatically declining [1]. Due to the construction of hydropower dams, the number of large free-flowing rivers on Earth will be reduced by approximately 21% in 2040 [2]. Overfishing is extensive in the developing world, and numerous fish populations have rapidly declined due to intensive fishing [3]. Large and valuable target species have been replaced by small and low-value fish species, which is called the fishing down process [4]. The present fish extinction rates are exceptionally high [5]. Therefore, the conservation of freshwater fish populations is receiving increasing attention [6–8].

Protected areas are a cornerstone of biological conservation [9]. Globally, the sharp increase in the number and extent of marine protected areas (MPAs) over the last few decades has contributed to increases in fish abundance and has alleviated the impacts of fishing on marine ecosystems [10, 11]. In contrast to the conservation practices of marine environments, the use of freshwater protected areas (FPAs) for the conservation of freshwater environments has been relatively limited [12–14]. In addition, many FPAs are embedded within terrestrial protected areas with underlying objectives that focus on terrestrial conservation and are therefore disconnected from freshwater issues [15]. Doubts regarding the benefits of the existing protected areas for freshwater conservation have arisen since few of these protected areas have been studied [6]. Given the experience in the marine realm, researchers and FPA practitioners are learning from their MPA counterparts [16].

The main methods used in previous studies to determine the effects of FPAs include self-contained underwater breathing apparatus (SCUBA) surveys [8], traditional catching [6, 17, 18], and satellite imagery [19]. Large female fish are far more productive than the same weight of small females [20]. Moreover, the older individuals of some fish species produce larvae that have substantially better growth, larger sizes, and higher survival rates than larvae from younger fish, and large fish usually have exponentially higher fecundity than small fish [21, 22]. Although older or larger individuals in fish populations might be crucial for the maintenance of stock resources and genetic heterogeneity, this aspect has not received due attention [21, 23]. Little is known about the temporal and spatial patterns of mature fish species in FPAs since these patterns are difficult to determine using traditional techniques. The development and improvement of scientific acoustic instruments over the last decade has enabled the precise characterization of the spatial distribution and abundance of fish, whereas traditional shallow-water netting techniques are difficult to implement [24, 25].

Systematic conservation planning (SCP) is a critical approach for designing a regional reserve network [26]. Currently, some freshwater conservation planning efforts use software packages such as Marxan [27] and C-Plan [28] to guide decisions regarding the selection of conservation areas. Marxan has been reported to greatly increase biodiversity conservation efficiency around the world [14, 29–32].

**Fig. 1:**
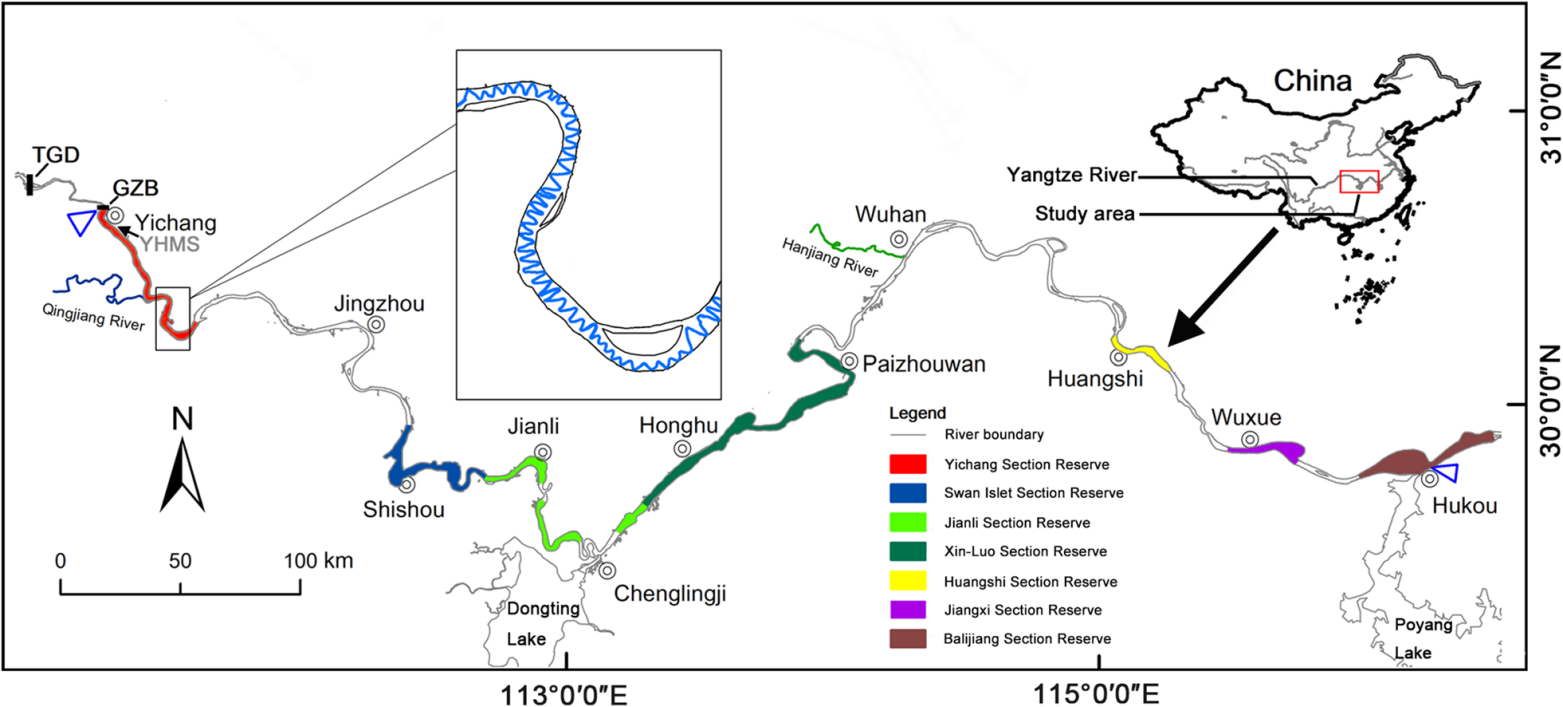
Study area located from the Gezhouba Dam to Hukou in the Yangtze River, China, a river length of approximately 906.7 km. The two small blue triangles show the start and end of the study area. In the expanded box, the zigzag line shows the route of the acoustic survey. YHMS: Yichang Hydrological Monitoring Station

The Yangtze River is the third largest river in the world and is highly impacted by human activities. The fishes in the Yangtze River are essential for the sustainable development of freshwater fisheries and the conservation of aquatic biodiversity in China [33]. The middle reach of the Yangtze River (MRYR, from Yichang to Hukou, ~ 900 km long) (Fig. 1), which is characterized by the connection of the river channel to floodplain lakes, has historically been a major freshwater fishery area in China [34]. This region is also one of the richest areas in terms of freshwater fish species diversity, with approximately 215 fish species, 42 of which are endemic [33, 35]. However, the survival rate of those fish species in this region has been drastically reduced due to human activities [36]. The rise in water temperature resulting from the impoundment of the Three Gorges Dam (TGD) caused habitat degradation and delays in the spawning of Chinese sturgeon and the four major Chinese carp species [37–39]. In addition, habitat modification or fragmentation, introduction of exotic species, overexploitation of resources, inbreeding depression of the four major Chinese carp species, and deterioration of ecological environments due to pollution appear to be the most serious threats to the fish stocks in the MRYR [40, 41]. To protect rare and economically critical aquatic animals in this region, 3 nature reserves and 4 germplasm resource reserves were established by the Chinese government (with their features shown in Fig. 1 and Table 1). The protected areas covered 50.23% of the watercourse length in the MRYR. The nature reserves in this area focus mainly on the conservation of rare aquatic animals and their habitats, such as *Acipenser sinensis*, *Lipotes vexillifer*, and *Neophocaena phocaenoides asiaeorientalis* [42], whereas the germplasm resource reserves focus mainly on the protection of the germplasm resources of economically important species, such as the four major Chinese carp species, *Leiocassis longirostris*, and *Silurus asotus*. Generally, the reserves are divided into three parts: the core area, buffer zone, and experimental zone. Moreover, these reserves are legally enforced. The ranges and boundaries of the reserves were determined after considering the integrity and suitability of the protected resources as well as the needs of the local economy, the production activities, and the everyday lives of the residents in the area [43, 44]. However, nature reserves and germplasm resource reserves that regard freshwater fishes as the main object of protection are less common and have been less studied in China [36, 45]. Considering its importance in the Yangtze River ecosystem, the MRYR was selected to evaluate the effects of these types of reserves. In this study, hydroacoustic technology and Marxan were combined to investigate the effectiveness of reserves based on the spatial and temporal patterns of mature fishes in the MRYR. The results could provide guidance for improving the management efficiency of the reserves and promoting the conservation of fish populations in the MRYR.

**Table 1:** Features of reserves and non-reserves in the middle reach of the Yangtze River

Protected area	Location ID	Type of reserve	Year establishment	Age (y)	Watercourse length (km)	Total surface (ha)	Main Objects to protect
Chinese Sturgeon (*Acipenser sinensis*) Reserve in the Yichang Reach of the Yangtze River	S1	Nature reserve	1996	21	80.0	8000	Chinese sturgeon
Islet Section Reserve: National Nature Reserve of Baiji (*Lipotes vexillifer*) in the Swan Islet of the Yangtze River*	S3	Nature reserve	1990	27	89.0	13,350	Baiji dolphin; Yangtze finless porpoise; Chinese sturgeon
National Germplasm Resource Reserve of the Four Major Chinese Carps in the Jianli section of the Yangtze River	S4, S6, S8	Germplasm resource reserve	2010	7	78.5	15,996	The four major Chinese carp species
National Nature Reserve of Baiji (*Lipotes vexillifer*) in the Xin-luo section of the Yangtze River	S9	Nature Reserve	1992	25	135.5	41,387	Baiji dolphin; Yangtze finless porpoise; Chinese sturgeon
National Germplasm Resource Reserve of the Four Major Chinese Carps in the Huangshi section of the Yangtze River	S11	Germplasm resource reserve	2008	9	26.5	4094	The four major Chinese carp species
National Germplasm Resource Reverse of the Four Major Chinese Carps in the Jiangxi section of the Yangtze River	S13	Germplasm resource reserve	2016	1	26.0	2725	The four major Chinese carp species; Long-snout catfish; catfish
National Germplasm Resource Reserve of Longsnout Catfish (*Leiocassis longirostris* Gunther) and Southern Catfish (*Silurus meridionalis* Chen) in the Balijiang section of the Yangtze River	S15	Germplasm resource reserve	2015	2	23.5	7993	Long-snout catfish; catfish
Non-reserve	S2, S5, S7, S10, S12, S14						

The total surface of the mainstream of the Yangtze River in the Swan Islet section reserve* has not been strictly determined by the government; therefore, a value of 13,350 ha was used based on the product of the total length of the river boundary (89 km) and the mean width of the river (1.5 km)

## Results

### River environment

**Fig. 2:**
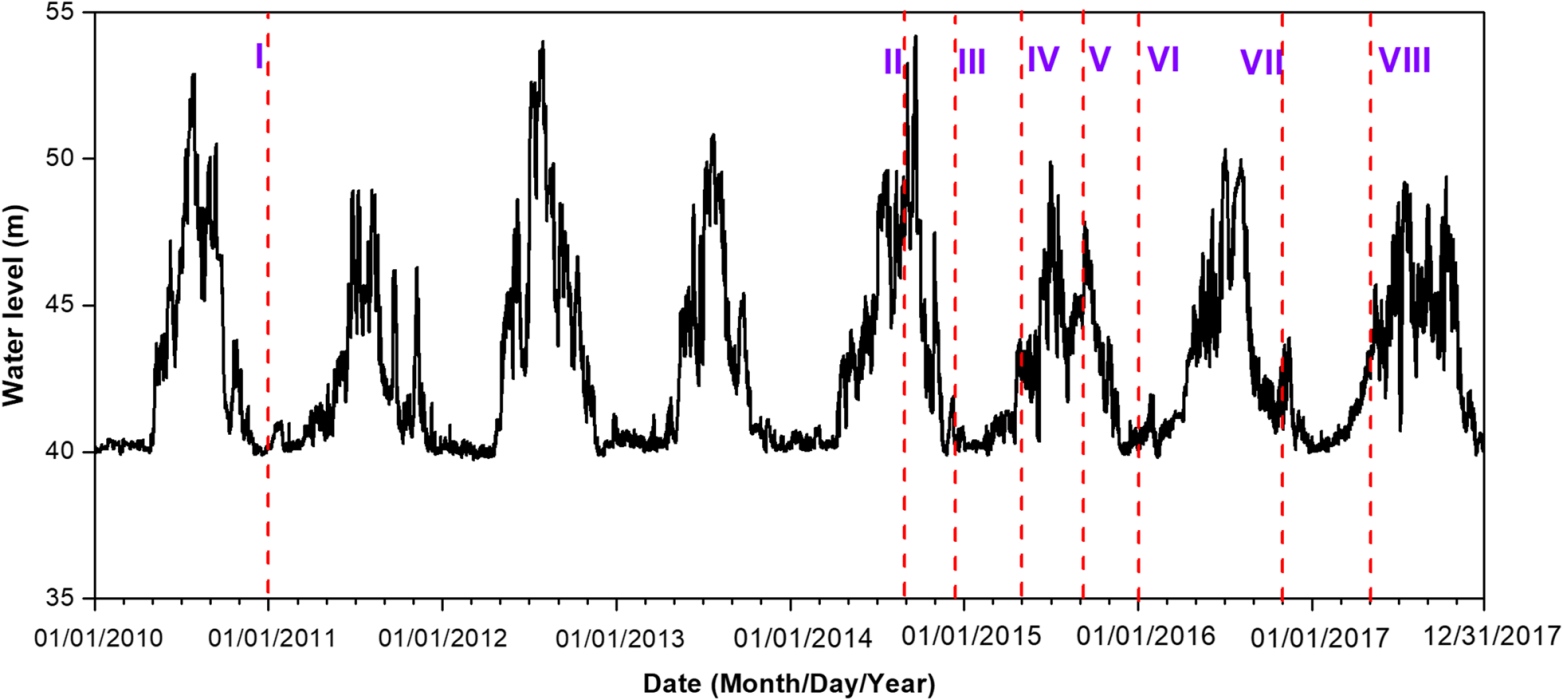
Profiles of water levels in the 906.7 rkm river reach downstream of the Gezhouba Dam, Yangtze River, 2010–2017, surveyed by the YHMS (Yichang Hydrological Monitoring Station). The vertical dotted line represents the date of the hydroacoustic survey; Roman numerals represent the survey IDs (Fig. 1; Table 3)

The water level at the Yichang Hydrological Monitoring Station (YHMS) during the acoustic surveys is presented in Fig. 2. The mean level was 42.9 m from 2010 to 2017. The water level peaked from July to September (mean 46.12 ± se 0.73 m) and then declined from January to March (mean 40.52 ± se 0.11 m) when the lowest water levels were measured. Consequently, the year (2017) was divided into four seasons: low water (January–March), rising water (April–June), high water (July–September), and falling water (October–December) based on the water level at the YHMS [18].

### Fish assemblages

A total of 30 761 fishes belonging to 9 orders, 19 families, 61 genera, and 106 species were acquired (Additional file 1). Of the 106 species, 29 species, each of whose percentages (%N) exceeded 0.5% of the total number of catches (30,761), accounted for 92.49% of the total number and 91.97% of the total weight of all catches. The fish fauna of all catches consisted mostly of Cyprinidae (64.24% of all taxa), Bagridae (16.97%), Serranidae (12.41%), and Clupeidae (2.5%).

### Acoustic target identification of large individuals

The numbers of mature individuals (target strength, TS > − 42.5 dB) analyzed in eight acoustic surveys were 147, 393, 61, 215, 89, 74, 306, and 88 in the 2010 low water, 2014 high water, 2014 low water, 2015 rising water, 2015 high water, 2015 low water, 2016 low water, and 2017 rising water seasons, respectively.

### Fish spatial–temporal distribution

**Fig. 3:**
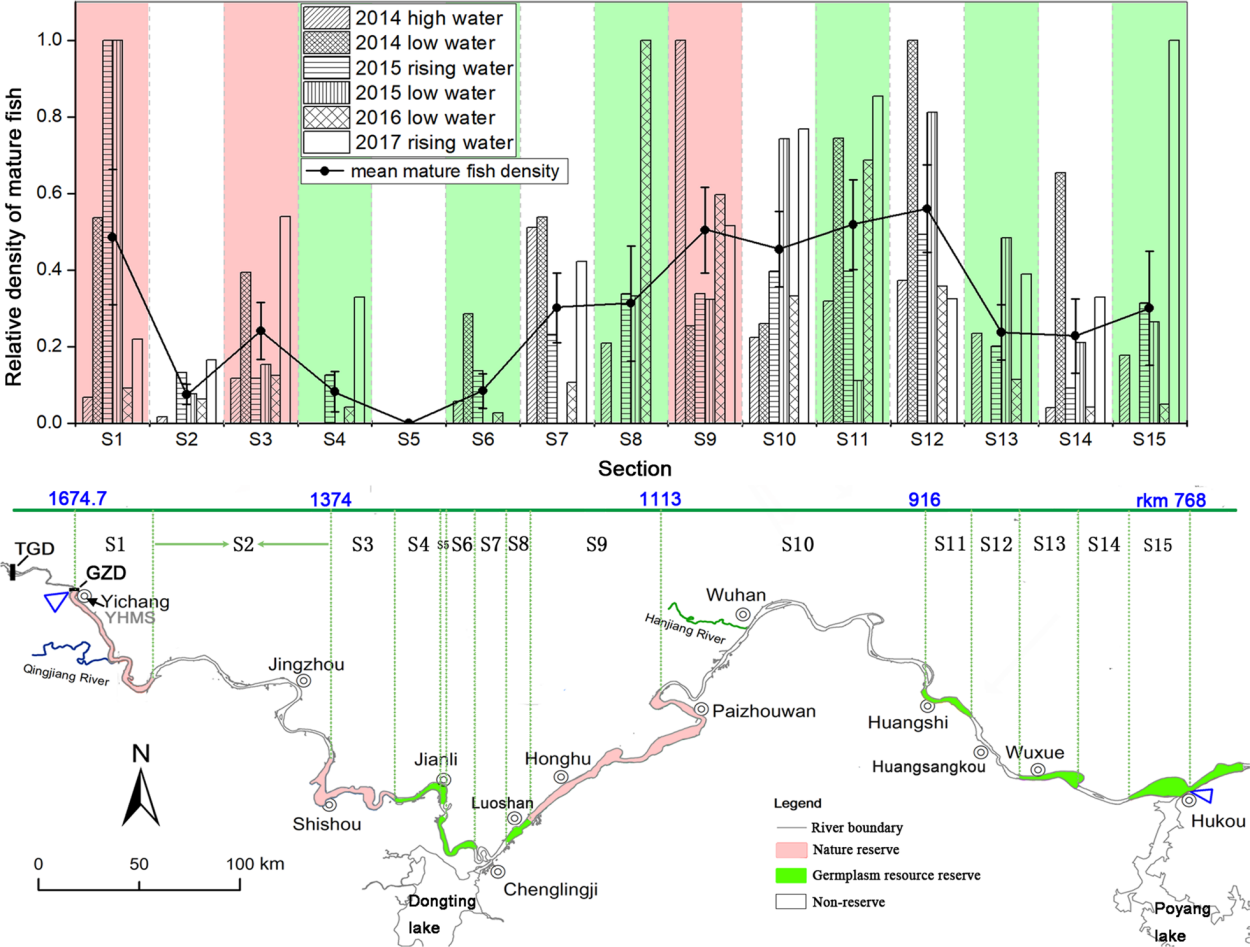
Longitudinal fish distribution in different sections of the middle Yangtze River. Fifteen sections were delimited based on the location of 7 freshwater protected areas; the data from the 2010 low water period and 2015 falling water period were not used to calculate the mean density of large fish because the surveys were terminated at the Wuhan section (Table 3)

The distribution of mature individuals in the longitudinal direction was displayed by the relative fish density (*density′*). The acoustic survey was conducted along a length of 906.7 km, and the river was divided into 15 reaches (S1–S15) according to the locations of the reserves (Fig. 3). In the longitudinal direction, low densities of mature fish were observed in the Jingzhou (S2, *density′ *= mean 0.08 ± se 0.03) and Jianli (S4, *density′ *= mean 0.08 ± se 0.05; S5, *density′ *= mean 0; and S6, *density′ *= mean 0.08 ± se 0.05) sections, whereas high densities of mature fish were found in other sections, such as the Yichang (S1, *density′ *= mean 0.49 ± se 0.18), Chenglingji (S7, *density′ *= mean 0.30 ± se 0.09), Luoshan (S8, *density′ *= mean 0.31 ± se 0.15), Xin-luo (S9, *density′ *= mean 0.50 ± se 0.11), Wuhan (S10, *density′ *= mean 0.45 ± se 0.10), Huangshi (S11, *density′ *= mean 0.52 ± se 0.12), Huangsangkou (S12, *density′ *= mean 0.56 ± se 0.11), and Hukou (S15, *density′ *= mean 0.30 ± se 0.15) sections. Among the regions with high mature fish densities, S7, S10 and S12 are located in non-reserve areas.

**Fig. 4:**
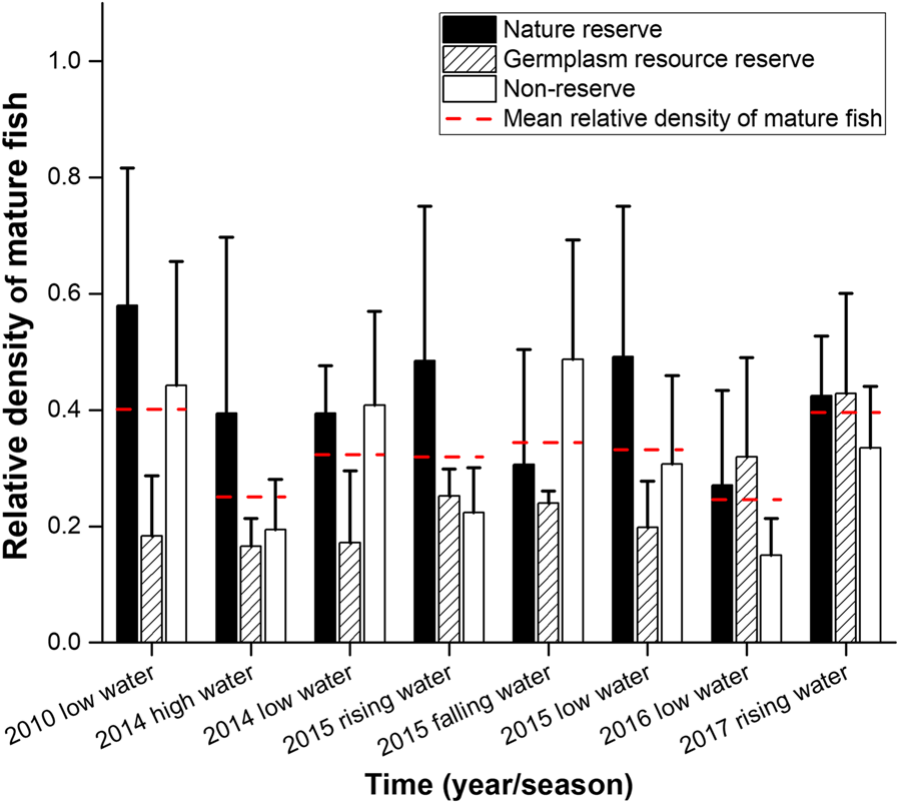
Relative density of mature fish (target strength > − 42.5 dB) in the nature reserves, germplasm resource reserves, and non-reserves during the acoustic detection survey from 2010 to 2017

No significant differences in the density of large fish were found among the nature reserves, germplasm resource reserves, and non-reserves when each acoustic survey result was examined individually (p > 0.05) (Fig. 4). When the eight acoustic detection survey results were examined together, the relative density of mature fish in the non-reserves (*density′ *= mean 0.32 ± se 0.04) was lower than that in the nature reserves (*density′ *= mean 0.42 ± se 0.04) but higher than that in the germplasm resource reserves (*density′ *= mean 0.25 ± se 0.03). However, these differences were not statistically significant. As a paired *t* test showed, no significant difference in mature fish density was observed between the non-reserves and nature reserves (t = 1.781, p = 0.097), and a similar result was obtained between the non-reserves and germplasm resource reserves (t = − 1.368, p = 0.193).

The overall density of mature fish in the MRYR increased from the 2014 low water period to the 2015 low water period, but this increase was not significant. However, the increase in the density became significant from the 2015 low water period to the 2016 low water period (p < 0.05) and from the 2014 low water period to the 2016 low water period (p < 0.01) (Table 2). In the nature reserves, the density of mature fish increased from the 2014 low water period to the 2015 low water period and from the 2015 low water period to the 2016 low water period, but these increases were not statistically significant. There was a significant increase in the density from the 2014 low water period to the 2016 low water period. Similar results were observed for the germplasm resource reserves. The density increased in the non-reserves, but this increase was not significant (p > 0.05) (Table 2).

**Table 2:** Mann-Whitney U test results of the density of mature fish in nature reserves, germplasm resource reverses and non-reserves, in the low water period from 2014 to 2016

Area	2014 low water (m^−3^)	2015 low water (m^−3^)	2016 low water (m^−3^)
Nature reserves	1.86 × 10^−6^ ± 3.86 × 10^−7 a^	3.42 × 10^−6^ ± 1.79 × 10^−6 ab^	9.52 × 10^−6^ ± 5.73 × 10^−6 b^
Germplasm resource reserves	8.08 × 10^−7^ ± 5.82 × 10^−7 a^	1.38 × 10^−6^ ± 5.252 × 10^−7 ab^	1.13 × 10^−5^ ± 6.01 × 10^−6 b^
Non-reserves	1.92 × 10^−6^ ± 7.60 × 10^−7 a^	2.13 × 10^−6^ ± 21.06 × 10^−6 a^	5.28 × 10^−6^ ± 2.23 × 10^−6 a^
Total	1.46 × 10^−6^±3.95 × 10^−7 a^	2.09 × 10^−6^ ± 5.79 × 10^−7 a^	8.52 × 10^−6^ ± 2.71 × 10^−6 b^

With respect to the different years, different letters indicate a significant difference among areas in the middle reach of the Yangtze River (p < 0.05)

### FPA optimization

**Fig. 5:**
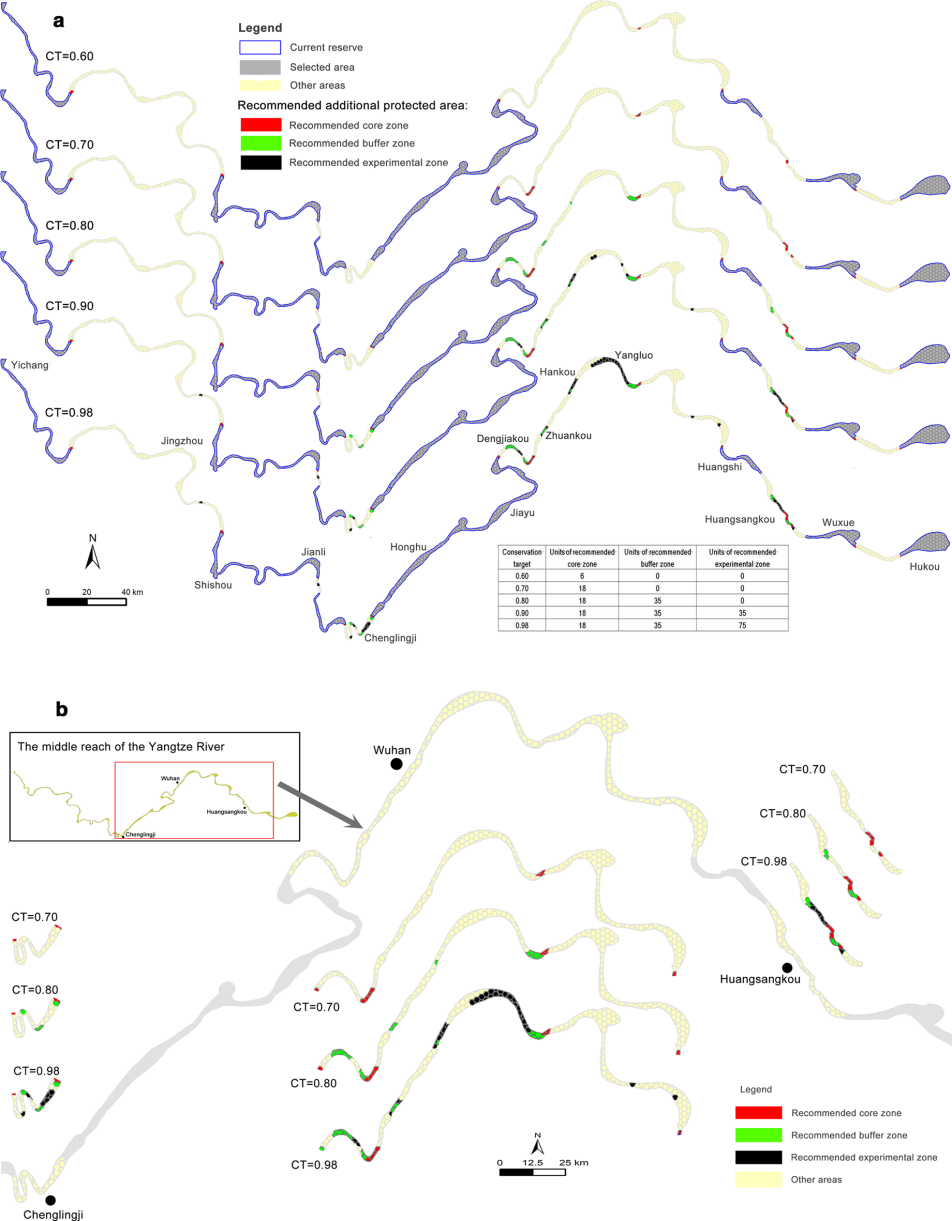
**a** Optimal selected conservation areas under 5 scenarios with different conservation targets (CTs) (0.60, 0.70, 0.80, 0.90 and 0.98). In the inserted table, the number of planning units for the recommended core zone, buffer zone, and experimental zone corresponding to different CTs are presented. **b** An enlarged drawing of the recommended core zone, buffer zone and experimental zone in non-reserves (Chenglingji, Wuhan and Huangsangkou sections)

**Fig. 6:**
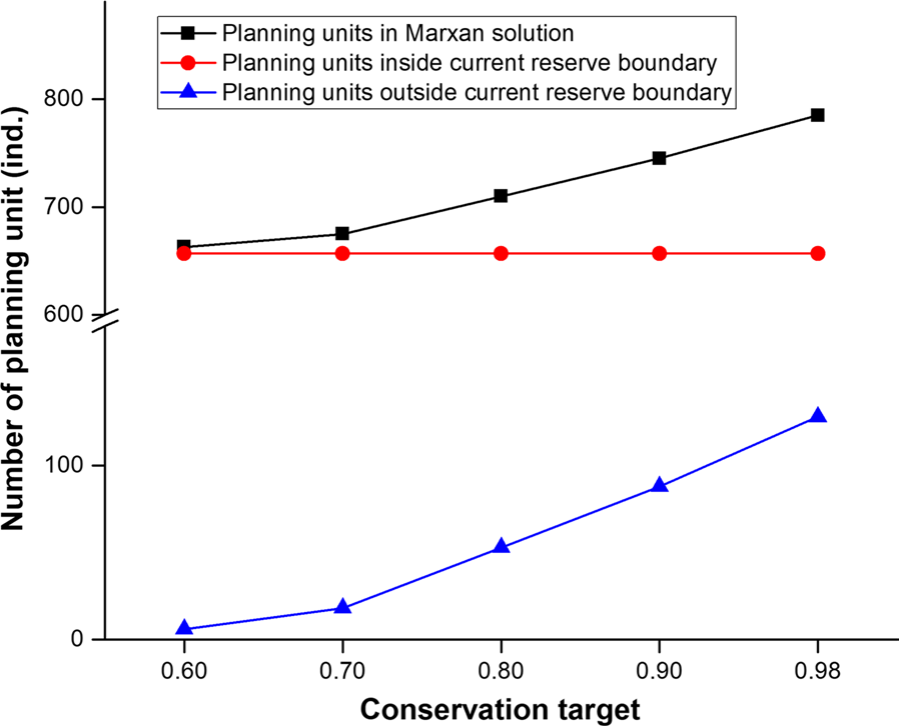
Number and distribution of planning units with different conservation targets (CTs) (0.60, 0.70, 0.80, 0.90 and 0.98)

According to 5 scenarios with different conservation targets (CTs) (0.60, 0.70, 0.80, 0.90, and 0.98), Marxan selected some optimal conservation sites in the MRYR (Fig. 5a). A positive correlation was found between the different CTs and the number of planning units (PUs) reaching the protection goal (R^2^ = 1, p < 0.01) (Fig. 6). When CT = 0.98, 128 PUs were selected outside the current reserves as the optimal protected areas. When CT = 0.60, only 6 PUs were found outside the current reserves. Optimal conservation sites for habitat restoration, such as the Chenglingji, Dengjiakou, Zhuankou, Hankou, Yangluo, and Huangsangkou sections located in non-reserves, were identified and selected. Protection measures should be preferentially implemented in these areas (Fig. 5).

## Discussion

This research indicated that the combination of hydroacoustic technology and Marxan was feasible to investigate the effectiveness of reserves based on the spatial and temporal patterns of mature fishes in the MRYR. In addition, Marxan selected optimal conservation areas that were non-reserves in the MRYR, such as the Chenglingji, Dengjiakou, Zhuankou, Hankou, Yangluo, and Huangsangkou sections (Fig. 5).

However, there are some limitations in this study. For example, fish sampling could not be conducted from April 1st to June 31st before 2015 or from March 1st to June 30th after 2016, when fishing was banned. Many previous studies measured the catch per unit effort (CPUE), the abundance of endemic species, and biodiversity and used these measurements as indicators to evaluate the effectiveness of reserves [18, 46, 47]. However, it is inappropriate to use these factors in this particular region because it is difficult to sample fish in the turbulent, uneven, and gravel environment of the MRYR. Hydroacoustic and trawl surveys are often conducted simultaneously in oceanic environments [48]. However, the complex physical environment of the river and heavy shipping in this region make synchronic acoustic detection and fish sampling extremely difficult. In addition, noise echo signals produced by the turbulent flow environment (especially in the high water seasons) further restricted acoustic sampling. However, acoustic sampling is still considered a useful and nonintrusive technique that can provide long-term observations in fishery research [49].

We obtained the optimal conservation sites in non-reserves from Marxan. High mature fish density was found in non-reserves. These results indicated that the conservation gaps for fish species were obvious.

The optimal solution is a typical representation of protected areas with comprehensiveness, adequacy, representativeness, and efficiency in the MRYR. Regarding the optimal solution in Marxan, 16.3% of PUs were found in non-reserves when CT = 0.98. This result indicates that there is an obvious deficiency in the existing fish reserve network. The Chenglingji, Dengjiakou, Zhuankou, Hankou, Yangluo, and Huangsangkou sections, which were selected as optimal conservation sites in Marxan, are located in non-reserve areas (Fig. 5).

In our study, no significant difference in mature fish density was observed between non-reserves and nature reserves, and a similar result was also observed between non-reserves and germplasm resource reserves. Additionally, the Chenglingji (S7), Wuhan (S10), and Huangsangkou (S12) sections, which had high mature fish density, were located in non-reserves. Therefore, the survey results of the relative density of mature fish were consistent with the results of Marxan (Figs. 3, 5).

The interannual variations in the density of mature fish during the low water periods in 2014, 2015, and 2016 were analyzed by the Mann–Whitney U test. The overall density of mature fish increased from 2014 to 2015, but such an increase was not statistically significant, whereas a significant increase was observed from 2015 to 2016 (p < 0.05), and a highly significant increase from 2014 to 2016 (p < 0.01) was observed. We inferred that the sharp increase might have been attributed to exotic sturgeon that escaped from farm cages in the Qingjiang River, a branch of the Yangtze River in Hubei Province, during a flood discharge on July 19th, 2016. In our fish sampling investigation, a high number of hybrid sturgeons (N = 40, total length = 105.6 ± 41.6 cm; body weight = 7696.6 ± 8753 g) and one Russian sturgeon (N = 1, total length = 128 cm; body weight = 8672 g) were found in the Yichang (S1), Jingzhou (S2), Shishou (S3), Honghu (S9), and Qizhou (S12) sections, and most of these individuals (N = 30) were obtained immediately below the Gezhouba Dam (GZD), which is the last remaining spawning ground for Chinese sturgeon. Invasive sturgeons and the rare Chinese sturgeon have identical niches. Consequently, there might be a competition between these species, leading to an unfavorable impact on the survival of Chinese sturgeon. A hybridization risk also exists between nonnative sturgeons and Chinese sturgeon, which may result in a decrease in purebred germplasm genes and reduced biotic fitness of the endangered Chinese sturgeon. Table 2 shows that the density of fish in the nature reserve and germplasm resource reserves increased significantly (p < 0.05), while no significant increase was observed in the non-reserves. This result indicates that the escaped sturgeon might prefer to stay inside the reserves, which should be further investigated.

There are many reasons for the poor effectiveness of FPAs: lack of adequate consideration of freshwater needs when designing and determining protected areas, lack of sufficient resources devoted to freshwater conservation management, and a poor understanding of complex management problems beyond the limits of the protected area [12]. Our findings indicated that further optimization urgently needs to be performed for the current protected areas in the MRYR. Previous studies have shown that some non-reserves also play equally important roles in the conservation of fish populations [50]. Therefore, we suggest that the optimal conservation sites selected by Marxan should be earmarked as reserves to strengthen the effectiveness of fish conservation in the MRYR.

High CT requires the creation of more protected units. However, in practice, conservation funds are often limited. Too many or too few protection units will result in reduced efficiency. This study provides a solution to the problem of choosing the protection units with the highest protection efficiency under the conditions of limited funds for decision makers. However, in the case of a limited amount of funds, equal allocation of funds into all protection units often does not achieve the best effect in practical applications. Thus, it is recommended to designate a core zone (selected small areas under low CT), buffer zone (selected small areas under high CT), and experimental zone (other optimal selected areas) (Fig. 5) with the allocated funds, with preference given to the core zone to ensure the better protection of this zone under this classification protection mechanism. In our study, the CT values of the core zone, buffer zone, and experimental zone were set as 0.70, 0.80, and 0.98, respectively. The parameters may be adjusted according to the actual situation.

Our results indicated that the combination of hydroacoustic detection and Marxan is feasible to improve the efficiency of FPAs for fish conservation in the main stem sections of large rivers. Additionally, the effectiveness of freshwater biodiversity conservation remains to be enhanced in subcatchments [30, 51]. In the future, more efforts should focus on connectivity-driven key ecological processes [52], aquatic refugees [19], and threat mitigation [53].

## Conclusions

The results of this study indicated that the combination of hydroacoustic detection and a SCP tool is a feasible way to increase the effectiveness of FPAs for fish conservation in the main stem of the MRYR. We confirmed that some optimal conservation sites such as those in the Chenglingji, Dengjiakou, Zhuankou, Hankou, Yangluo, and Huangsangkou sections in non-reserves played roles in the protection of fish populations in this region that were equally important to those played by the reserves. A better understanding of the distribution of mature fish can help resource managers and policy makers design better FPAs in response to specific management problems. Our findings are important and necessary for the improvement of protection effectiveness. Many other aspects should be further investigated, such as socioeconomic factors, enforcement of local governments, periodic harvest closures, and the establishment of a protected network, which would benefit the conservation of fish populations in the MRYR.

## Methods

### Study area

The study area covers the main stem of the MRYR (Fig. 1) from downstream of the GZD (river kilometer (rkm) 1674.7 km) to Hukou (rkm 768 km), with a length of approximately 906.7 km (the Yangtze River estuary was defined as rkm 0). The largest (Poyang Lake) and second largest (Dongting Lake) Chinese freshwater lakes intersect with the Yangtze River in Chenglingji and Hukou, respectively. The geomorphology of the MRYR varies obviously from the mountains to the more alluvial river section, and the hydrological characteristics are regulated by the GZD and the TGD (located 38 km upstream of the GZD). This region is the habitat and breeding grounds for species with drifting eggs, and it is also the migration channel for endangered and rare species, such as Chinese sturgeon (*Acipenser sinensis* Gray 1835) and Chinese paddlefish (*Psephurus gladius* Martens 1862). The downstream area of the GZD is the only remaining spawning ground of Chinese sturgeon [39, 54]. Twenty-seven spawning grounds of the four major Chinese carp species identified in this region accounted for 75% of the total number of spawning grounds. These 27 spawning grounds produced approximately 69% of the total offspring in the main stem of the Yangtze River [35, 55].

### Fish sampling

Fishing with various gillnets (2, 6, 8 cm) and shrimp pots was carried out to obtain catches in each sampling section. Fixed gillnets (20 mm mesh size) were placed 50–100 m offshore, and shrimp pots were positioned 10–20 m offshore in the evening (18:00–06:00) each day. Drift nets (60 mm mesh size; 80 mm mesh size) were used in deep water during the day from 6:00 to 18:00. The catches were acquired from July 1st–20th, 2015, November 5th–25th, 2015, November 5th–25th, 2016, and July 1st–20th, 2017. The six sampling sections were denoted as S1, S2, S8, S9, S10, and S13 (Fig. 3). During the closed season lasting from March 1st to June 31st, catches cannot be obtained. Fish were captured continuously for 20 days per site using various types of nets in summer and winter in various habitats, including pools, riffles, backwaters, and runs. The phylogenetic classification of fish was conducted as described in previous studies [56–59]. The body length (in millimeters) and weight (in grams) of each species were measured after the fish were anesthetized in MS-222 (Sigma). The fishes protected by the Chinese government and those threatened species on the IUCN (International Union for the Conservation of Nature and Natural Resources) and CITES (Convention on International Trade in Endangered Species of Wild Fauna and Flora) lists were released back into the river, and the commercial species were returned to fishermen.

### Hydroacoustic surveys

Eight acoustic detection surveys were carried out in different seasons from 2010 to 2017 (Table 3). A SIMRAD EY60 split-beam echo sounder (SIMRAD, Norway) with a frequency of 200 kHz and an opening angle of 7° at − 3 dB was used. Hydroacoustic measurements were conducted using a 6.4 m long fiberglass-reinforced plastic boat with a 120 HP outboard engine operating at a speed of 8–10 km h^−1^. The survey route followed dense zigzag lines (Fig. 1).

**Table 3:** Summary of eight acoustic surveys conducted in MRYR from 2010 to 2017

Year		Survey ID		Sampling date			Mean water level* (m)		Season		Survey period (d)	River stretch (from-to)		Start-endpoint (rkm)
2010/2011		I		Dec 18, 2010–Mar 5, 2011			40.31		Low water		37	Yichang–Wuhan		1674.7–1038
2014/2015		II		Aug 13, 2014–Sept 9, 2014			48.15		High water		32	Yichang–Hukou		1674.7–768
		III		Dec 18, 2014–Feb 7, 2015			40.28		Low water		36	Yichang–Hukou		1674.7–768
2015		IV		Apr 25, 2015–Jun 6, 2015			42.64		Rising water		33	Yichang–Hukou		1674.7–768
		V		Sept 11, 2015–Oct 20, 2015			44.80		Falling water		25	Yichang–Wuhan		1674.7–1038
2016		VI		Jan 9, 2016–Feb 26, 2016			40.69		Low water		31	Yichang–Hukou		1674.7–768
		VII		Nov 22, 2016–Jan 2, 2017			40.53		Low water		44	Yichang–Hukou		1674.7–768
2017		VIII		May 14, 2017–Jul 17, 2017			45.01		Rising water		30	Yichang–Hukou		1674.7–768

Mean water level* is the mean water level determined by the Yichang Hydrological Monitoring Station (YHMS)

The transducer was aimed vertically downward and was anchored on the right side of the boat on a special frame at a depth of 0.5 m to sample the entire water column from 1 m below the water surface to 0.5 m above the bottom of the water body. During detection, the pulse duration was set as 256 us with a power output of 180 W, and the repetition rate was as fast as possible. The TS threshold was set as − 80 dB, which was the lowest possible value that did not include too much noise in the echogram. The echo sounder was connected to a portable computer, which provided a real-time display and data storage. The geographical positions of the soundings were recorded simultaneously by a global positioning system (Garmin, Taiwan, China) connected to the sounder. At the beginning of each survey, the whole system was calibrated in situ according to the SIMRAD instruction manual. All detections were performed in the daytime from 8:30 to 16:30.

### River environment measurements

The water level data were obtained from the YHMS. The other hydrological data for the MRYR were derived from the literature [60–62].

### Size determination of mature fish

The first 29 fish species (N % > 0.5%, Total N % = 92.49%, Total W % = 91.97%) that were ranked in terms of percentages were used to determine the mature fish (Additional file 1). The median initial mature body length (BL = 13.5 cm) of these species was determined. BL was converted into TS (TS = − 42.5 dB) using the Love formula, which is widely applied to estimate fish TS when accurate TS information is lacking [63].

### Hydroacoustic data analysis

S_ONAR_-5 P_RO_ software (University of Oslo, Oslo) was used to process and analyze the echo sounder data [64]. Only the data from 1 m beneath the water surface to 0.5 m above the river bottom were used in the analysis. Four main procedures were used to determine the targets [65]. (I) File conversion: the raw data files (.raw) were converted into.uuu files by the converter in S_ONAR_-5 P_RO_. (II) Bottom detection: the bottom detector (image analysis detector) determined the river bottom line in each file. Manual rectification was conducted to improve the bottom line developed by the detector. (III) Target tracking: a multiple target tracker (MTT) was used to detect a single target, and optimal parameter settings were used. (IV) Track filtering: in the acquired fish-basket, track filtering was used to filter the targets with No. Echoes ≥ 4, − 42.5 dB ≤ Mean (TS) ≤ − 16 dB, Max Ping Gap = 2 ping, Gating Range = 0.3 m.

The density of mature individuals was estimated by the following formula:

1 $${\text{Density}} = {\text{target number}}/{\text{survey volume}}.$$

The target number was obtained as described above. The water volume in the survey was calculated as a triangular prism using the following formula:

2 $${\text{Survey volume}} = 0. 5 { } \times \, \left( { 2\times {\text{H}} \times {\text{tan3}}. 5^\circ \times {\text{H}}} \right) \, \times {\text{sampling pings }} \times 0. 4 2,$$

where H is the average water depth during the sampling period. The constant 0.42 was calculated from the average boat speed of 9 km h^−1^, which represents the average distance the boat covered during one ping.

The inconsistency in background noise caused sharp differences in signal extraction in each acoustic detection survey. We conducted preprocessing of the relative density of mature fish using Min–Max normalization to scale the values within a definite range. After normalization, the new density value between 0 and 1 was used for further analysis. Min–Max normalization as follows:

3 $$Density' = \frac{{Density - Min_{density} }}{{Max_{density} - Mix_{density} }},$$

where *density′* is the normalized value, *Min*_*density*_ and *Max*_*density*_ are the minimum and maximum density values of mature fish in each acoustic detection survey, respectively.

Statistical analyses were carried out using the following software packages: IBM SPSS Statistics 22 (IBM, USA) and Origin 2016 (OriginLab, USA).

### Protected area optimization

Marxan optimized a set of locations to meet the requirement of CT with minimum cost. The run of Marxan was set as simulated annealing, followed by iterative improvement. In the simulated annealing algorithm, the number of iterations and temperature decreases followed the default settings. Input data and other parameters were set as follows:

#### PUs

Due to the computational capabilities, the resolution of the grid cells was upscaled to 1 km × 1 km. This step resulted in 1255 PUs covering the mainstream of the MRYR. During the running of Marxan, the current reserves were locked into optimal selected conservation sites. Each PU had a unique identification code.

The selection of nature reserves and germplasm resource reserves was based on existing data from the State Forestry Administration (http://english.forestry.gov.cn/) and Ministry of Agriculture of PRC (http://english.agri.gov.cn/) (Fig. 1; Table 1). All the drafts of the reserves were digitalized in ArcGIS 10.2 (ESRI, 2013).

#### CTs

To find an adequate representation of conservation features, Marxan was run in 5 scenarios with different CTs (0.60, 0.70, 0.80, 0.90, and 0.98), with each scenario implemented for 100 runs. Considering that few mature fishes were detected in the hydroacoustic survey (see the Results section), the starting value of CT was set to 0.60.

#### Costs of PUs

According to the main sources of human disturbance on the MRYR, the cost of each PU was calculated based on five variables of disturbances. These disturbances involve hydrological changes (dam density and sluice density), water pollution (occurrence of oil refinery and density of chemical plants), and human development pressures (population density). The weight coefficients of variables and data sources referred to Huang et al. [36].

Marxan aims to find a minimized objective function to obtain an optimal solution. The function includes the cost of the selected sites and additional penalty (Eq. 4)

4 $${\text{Objective function}} = \sum {\text{Cost}} + {\text{SPF}} \times {\text{Penalty}} + {\text{BLM}} \times \sum {\text{Boundary}} .$$

The species penalty factor (SPF) was used to weigh the species penalty if the CTs (large fish) were not met. The boundary length modifier (BLM) added weighted importance relative to the other components of the objective.

The selection frequency of each PU in the 100 runs was calculated. Grid cells with more than 80 runs (which had the minimum objective function value) were selected as the priority areas for conservation.

## Supplementary information

**Additional file 1.** List of fish species identified as occurring for all collecting sections in the middle reach of the Yangtze River.

## Data Availability

The datasets used and/or analyzed during the current study are available from the corresponding author on reasonable request.

## Abbreviations

BL: body length
BLM: boundary length modifier
CARE: comprehensiveness, adequacy, representativeness, and efficiency
CITES: convention on international trade in endangered species of wild fauna and flora
CPUE: catch per unit effort
CT: conservation target
FPAs: freshwater protected areas
GZD: Gezhouba Dam
IUCN: international union for the conservation of nature and natural resources
MPAs: marine protected areas
MRYR: the middle reach of the Yangtze River
MTT: multiple target tracker
PHCs: periodically harvested closures
PU: planning unit
rkm: river kilometer, the Yangtze River estuary was defined as rkm 0
SCP: systematic conservation planning
SCUBA: self-contained underwater breathing apparatus
SE: standard error
SPF: species penalty factor
TGD: Three Gorges Dam
TS: target strength
YHMS: Yichang Hydrological Monitoring Station
%N: percentage number
%W: percentage weight

## References

[1] Dudgeon D, Arthington AH, Gessner MO, Kawabata Z-I, Knowler DJ, Leveque C, Naiman RJ, Prieur-Richard A-H, Soto D, Stiassny ML, Sullivan CA (2006). Freshwater biodiversity: importance, threats, status and conservation challenges. Biol Rev.

[2] Zarfl C, Lumsdon AE, Berlekamp J, Tydecks L, Tockner K (2015). A global boom in hydropower dam construction. Aquat Sci.

[3] Roberts CM, Hawkins JP (1999). Extinction risk in the sea. Trends Ecol Evol.

[4] Castello L, McGrath DG, Hess LL, Coe MT, Lefebvre PA, Petry P, Macedo MN, Reno VF, Arantes CC (2013). The vulnerability of Amazon freshwater ecosystems. Conserv Lett..

[5] Pimm SL, Jenkins CN, Abell R, Brooks TM, Gittleman JL, Joppa LN, Raven PH, Roberts CM, Sexton JO (2014). The biodiversity of species and their rates of extinction, distribution, and protection. Science.

[6] Chessman BC (2013). Do protected areas benefit freshwater species? A broad-scale assessment for fish in Australia’s Murray-Darling Basin. J Appl Ecol.

[7] McCusker MR, Curtis JMR, Lovejoy NR, Mandrak NE (2017). Exploring uncertainty in population viability analysis and its implications for the conservation of a freshwater fish. Aquat Conserv..

[8] Britton AW, Day JJ, Doble CJ, Ngatunga BP, Kemp KM, Carbone C, Murrell DJ (2017). Terrestrial-focused protected areas are effective for conservation of freshwater fish diversity in Lake Tanganyika. Biol Conserv.

[9] Giakoumi S, Scianna C, Plass-Johnson J, Micheli F, Grorud-Colvert K, Thiriet P, Claudet J, Di Carlo G, Di Franco A, Gaines SD (2017). Ecological effects of full and partial protection in the crowded Mediterranean Sea: a regional meta-analysis. Sci Rep..

[10] Mosqueira I, Cote IM, Jennings S, Reynolds JD (2000). Conservation benefits of marine reserves for fish populations. Anim Conserv.

[11] Pascal N, Brathwaite A, Brander L, Seidl A, Philip M, Clua E (2018). Evidence of economic benefits for public investment in MPAs. Ecosyst Serv..

[12] Hermoso V, Abell R, Linke S, Boon P (2016). The role of protected areas for freshwater biodiversity conservation: challenges and opportunities in a rapidly changing world. Aquat Conserv..

[13] Abell R, Allan JD, Lehner B (2007). Unlocking the potential of protected areas for freshwaters. Biol Conserv.

[14] Nel JL, Reyers B, Roux DJ, Cowlingc RM (2009). Expanding protected areas beyond their terrestrial comfort zone: identifying spatial options for river conservation. Biol Conserv.

[15] Suski CD, Cooke SJ (2007). Conservation of aquatic resources through the use of freshwater protected areas: opportunities and challenges. Biodivers Conserv.

[16] Loury EK, Ainsley SM, Bower SD, Chuenpagdee R, Farrell T, Guthrie AG, Heng S, Lunn Z, Al Mamun A, Oyanedel R, Rocliffe S, Satumanatpan S, Cooke SJ (2018). Salty stories, fresh spaces: lessons for aquatic protected areas from marine and freshwater experiences. Aquat Conserv..

[17] Kwik JTB, Yeo DCJ (2015). Differences in fish assemblages in protected and non-protected freshwater streams in a tropical urbanized country. Hydrobiologia.

[18] Keppeler FW, Hallwass G, Matias Silvano RA (2017). Influence of protected areas on fish assemblages and fisheries in a large tropical river. Oryx..

[19] Hermoso V, Ward DP, Kennard MJ (2013). Prioritizing refugia for freshwater biodiversity conservation in highly seasonal ecosystems. Divers Distrib.

[20] Barneche DR, Robertson DR, White CR, Marshall DJ (2018). Fish reproductive-energy output increases disproportionately with body size. Science.

[21] Birkeland C, Dayton PK (2005). The importance in fishery management of leaving the big ones. Trends Ecol Evol.

[22] Beldade R, Holbrook SJ, Schmitt RJ, Planes S, Malone D, Bernardi G (2012). Larger female fish contribute disproportionately more to self-replenishment. P Roy Soc B-Biol Sci..

[23] Berkeley SA, Chapman C, Sogard SM (2004). Maternal age as a determinant of larval growth and survival in a marine fish. Sebastes melanops. Ecology..

[24] Zhang H, Wang CY, Yang DG, Du H, Wei QW, Kang M (2014). Spatial distribution and habitat choice of adult Chinese sturgeon (*Acipenser sinensis* Gray, 1835) downstream of Gezhouba Dam, Yangtze River, China. J Appl Ichthyol..

[25] Tao J, Yang Z, Cai Y, Wang X, Chang J (2017). Spatiotemporal response of pelagic fish aggregations in their spawning grounds of middle Yangtze to the flood process optimized by the Three Gorges Reservoir operation. Ecol Eng.

[26] Margules CR, Pressey RL (2000). Systematic conservation planning. Nature.

[27] Game E and Grantham H. Marxan user manual: for Marxan version 1.8.10. Vancouver: University of Queensland; 2008.

[28] Pressey RL, Watts ME, Barrett TW, Ridges MJ, Moilanen A, Wilson KA, Possingham HP (2009). The C-Plan conservation planning system: origins, applications, and possible futures. Spatial conservation prioritization: quantitative methods and computational tools.

[29] Liang J, Gao X, Zeng GM, Hua SS, Zhong MZ, Li XD, Li X (2018). Coupling Modern Portfolio Theory and Marxan enhances the efficiency of Lesser White-fronted Goose’s (*Anser erythropus*) habitat conservation. Sci Rep..

[30] Hermoso V, Filipe AF, Segurado P, Beja P (2015). Filling gaps in a large reserve network to address freshwater conservation needs. J Environ Manage.

[31] Turak E, Linke S (2011). Freshwater conservation planning: an introduction. Freshwater Biol..

[32] Lawrence DJ, Larson ER, Liermann CAR, Mims MC, Pool TK, Olden JD (2011). National parks as protected areas for US freshwater fish diversity. Conserv Lett..

[33] Fu CZ, Wu JH, Chen JK, Qu QH, Lei GC (2003). Freshwater fish biodiversity in the Yangtze River basin of China: patterns, threats and conservation. Biodivers Conserv.

[34] Liu JK, Cao WX (1992). Fish resources of the Yangtze River basin and the tactics for their conservation. Res Environ Yangtze Basin..

[35] Song Y, Cheng F, Murphy BR, Xie S (2018). Downstream effects of the Three Gorges Dam on larval dispersal, spatial distribution, and growth of the four major Chinese carps call for reprioritizing conservation measures. Can J Fish Aquat Sci.

[36] Huang X, Li F, Chen J (2016). Reserve network planning for fishes in the middle and lower Yangtze River basin by systematic conservation approaches. Sci China Life Sci..

[37] Duan X, Liu S, Huang M, Qiu S, Li Z, Wang K, Chen D (2009). Changes in abundance of larvae of the four domestic Chinese carps in the middle reach of the Yangtze River, China, before and after closing of the Three Gorges Dam. Environ Biol Fish..

[38] Zhang H, Wu JM, Wang CY, Du H, Liu ZG, Shen L, Chen D, Wei QW (2016). River temperature variations and potential effects on fish in a typical Yangtze River reach: implications for management. Appl Ecol Environ Res.

[39] Wei QW, Ke FE, Zhang JM, Zhuang P, Luo JD, Zhou RQ, Yang WH (1997). Biology, fisheries, and conservation of sturgeons and paddlefish in China. Environ Biol Fish..

[40] Xie Y, Li ZY, Gregg WP, Dianmo L (2001). Invasive species in China—an overview. Biodivers Conserv.

[41] Chen D, Xiong F, Wang K, Chang Y (2009). Status of research on Yangtze fish biology and fisheries. Environ Biol Fish..

[42] Zhu P, Huang L, Xiao T, Wang J (2018). Dynamic changes of habitats in China’s typical national nature reserves on spatial and temporal scales. J Geogr Sci..

[43] Ministry of Agriculture of PRC. Interim administrative measures on aquatic germplasm resources conservation zones. 2011. PRC.

[44] State Forestry Administration. Byelaw of nature reserve of PRC. 1994. PRC.

[45] Wu RD, Possingham HP, Yu GZ, Jin T, Wang JJ, Yang FL, Liu SL, Ma JZ, Liu X, Zhao HW (2019). Strengthening China’s national biodiversity strategy to attain an ecological civilization. Conserv Lett..

[46] Hermoso V, Filipe AF, Segurado P, Beja P (2015). Effectiveness of a large reserve network in protecting freshwater biodiversity: a test for the Iberian Peninsula. Freshwater Biol..

[47] Turak E, Linke S, Abell R, Thieme M, Hermoso V, Finlayson M, Arthington A, Pittock J (2018). Defining and enhancing freshwater protected areas. Freshwater ecosystems in protected areas.

[48] McClatchie S, Thorne RE, Grimes P, Hanchet S (2000). Ground truth and target identification for fisheries acoustics. Fish Res.

[49] Simmonds J, Maclenan D (2005). Fisheries acoustics: theory and practice.

[50] Keith P (2000). The part played by protected areas in the conservation of threatened French freshwater fish. Biol Conserv.

[51] Nel JL, Roux DJ, Maree G, Kleynhans CJ, Moolman J, Reyers B, Rouget M, Cowling RM (2007). Rivers in peril inside and outside protected areas: a systematic approach to conservation assessment of river ecosystems. Divers Distrib.

[52] Hermoso V, Linke S, Prenda J, Possingham HP (2011). Addressing longitudinal connectivity in the systematic conservation planning of fresh waters. Freshwater Biol..

[53] Linke S, Turak E, Nel J (2011). Freshwater conservation planning: the case for systematic approaches. Freshwater Biol..

[54] Deng ZL, Yu ZT, Xu YG, Zhou CS (1985). Age determination and population structure of spawning Chinese sturgeon (*Acipenser sinensis* Gray). Acta Hydrobiol Sinica..

[55] Yi BL, Liang ZS, Yu ZT, Liang ZS (1988). The present situation of the spawning ground of the four Chinese domestic fishes in Changjiang (Yangtze River) after construction of the Gezhouba water control project. Gezhouba water control project and four famous fishes in Yangtze River.

[56] Ichthyologic Department of Hubei Province (1976). The fishes of Yangtze River.

[57] Chen YY (1998). Fauna Sinica. Osteichthyes, Cypriniformes II.

[58] Yue PQ (2000). Fauna Sinica. Osteichthyes, Cypriniformes III.

[59] Chu XL (1999). Fauna Sinica. Osteichthyes, Siluriformes.

[60] Zhang W, Yuan J, Han J, Huang C, Li M (2016). Impact of the Three Gorges Dam on sediment deposition and erosion in the middle Yangtze River: a case study of the Shashi Reach. Hydrol Res..

[61] Han J, Sun Z, Li Y, Yan Y (2017). Combined effects of multiple large-scale hydraulic engineering on water stages in the middle Yangtze River. Geomorphology.

[62] Zhou M, Xia J, Lu J, Deng S, Lin F (2017). Morphological adjustments in a meandering reach of the middle Yangtze River caused by severe human activities. Geomorphology.

[63] Love RH (1971). Dorsal-aspect target strenget of an individual fish. J Acoust Soc Am.

[64] Balk H. Sonar4 and Sonar5-Pro post processing systems: operator manual version 6.0.4. Lindem Data Acquisition A/S, Oslo, Norway. 2013. http://folk.uio.no/hbalk/sonar4_5/. Accessed 06 Oct 2018.

[65] Zhang H, Yang DG, Wei QW, Du H, Wang CY (2013). Spatial distribution and spawning stock estimates for adult Chinese sturgeon (*Acipenser sinensis* Gray, 1835) around the only remaining spawning ground during the trial operation of the newly constructed Three Gorges Project in the Yangtze River. China. J Appl Ichthyol..

