# Supplementary material for: Are river protected areas sufficient for fish conservation? Implications from large-scale hydroacoustic surveys in the middle reach of the Yangtze River
Source: BMC Ecol. 2019 Sep 25;19:42. doi: 10.1186/s12898-019-0258-4 (PMC6760103; doi:10.1186/s12898-019-0258-4)
Supplement: Supplementary file 1 — Additional file 1. List of fish species identified as occurring for all collecting sections in the middle reach of the Yangtze River. [file 12898_2019_258_MOESM1_ESM.docx]

**Additional file 1. List of fish species identified as occurring for all collecting sections in the middle reach of the Yangtze River.**

| **Species** | | | **Number**  **(N)** | **%N** | **Weight**  **(g)** | | | **%W** | | **Mean BL**  **(cm)** | | | **Mean initial mature BL (cm)**  **and data source** | | | |
| --- | --- | --- | --- | --- | --- | --- | --- | --- | --- | --- | --- | --- | --- | --- | --- | --- |
| *Coreius heterodon* (Bleeker) | | | 6532 | 21.23 | 1178175.8 | | | 18.97 | | 23.5 | | | 25.0 [1] | | | |
| *Siniperca chuatsi*(Basilewsky) | | | 3290 | 10.70 | 448686.0 | | | 7.22 | | 17.4 | | | 15.6 [2] | | | |
| *Pelteobagrus vachelli*(Richardson) | | | 2443 | 7.94 | 122775.9 | | | 1.98 | | 14.9 | | | 13.3 [3] | | | |
| *Hemiculter leucisclus* (Basilewsky) | | | 1740 | 5.66 | 28289.7 | | | 0.46 | | 9.3 | | | 6.0 [4] | | | |
| *Rhinogobio cylindricus* (Günther) | | | 1603 | 5.21 | 82529.1 | | | 1.33 | | 16.1 | | | 24.2 [2] | | | |
| *Parabramis pekinensis* (Basilewsky) | | | 1341 | 4.36 | 350083.5 | | | 5.64 | | 24.4 | | | 19.7 [1] | | | |
| *Leiocassis crassilabris* (Günther) | | | 975 | 3.17 | 31229.0 | | | 0.50 | | 12.1 | | | 10.6 [5] | | | |
| *Saurogobio dabryi* (Bleeker) | | | 951 | 3.09 | 23941.9 | | | 0.39 | | 12.8 | | | 10.6 [2] | | | |
| *Squalidus argentatus* (Sauvage et Dabry) | | | 936 | 3.04 | 11523.4 | | | 0.19 | | 9.3 | | | 4.7 [6] | | | |
| *Pelteobagrus nitidus* (Sauvage et Dabry) | | | 869 | 2.83 | 13548.2 | | | 0.22 | | 9.6 | | | 8.2 [7] | | | |
| *Xenocypris argentea* Günther | | | 818 | 2.66 | 75207.2 | | | 1.21 | | 17.1 | | | 15.7 [1] | | | |
| *Coilia brachygnathus* Kreyenherg et Pappenheim | | | 774 | 2.52 | 23495.8 | | | 0.38 | | 17.4 | | | 12.0 [2] | | | |
| *Rhinogobio typus* Bleeker | | | 769 | 2.50 | 81726.2 | | | 1.32 | | 20.0 | | | 19.6 [8] | | | |
| *Cyprinus carpio* Linnaeus | | | 688 | 2.24 | 957190.9 | | | 15.41 | | 33.9 | | | 18.9 [1] | | | |
| *Carassius auratus* (Linnaeus) | | | 622 | 2.02 | 120032.1 | | | 1.93 | | 15.3 | | | 12.7 [2] | | | |
| *Siniperca scherzeri* Steindachner | | | 486 | 1.58 | 81041.7 | | | 1.30 | | 20.6 | | | 11.8 [1] | | | |
| *Hypophthalmichthys molitrix* (Cuvier et Valenciennes) | | | 436 | 1.42 | 741113.8 | | | 11.93 | | 40.5 | | | 58.4 [2] | | | |
| *Pseudobrama simoni* (Bleeker) | | | 393 | 1.28 | 16528.0 | | | 0.27 | | 11.7 | | | 8.2 [9] | | | |
| *Squaliobarbus curriculus* (Richardson) | | | 360 | 1.17 | 114824.9 | | | 1.85 | | 27.7 | | | 23.5 [2] | | | |
| *Hemiculter bleekeri* Warpachowsky | | | 351 | 1.14 | 4866.4 | | | 0.08 | | 8.3 | | | 7.5 [10] | | | |
| *Pelteobagrus fulvidraco* (Richardson) | | | 295 | 0.96 | 14641.8 | | | 0.24 | | 13.2 | | | 9.7 [11] | | | |
| *Mystus macropterus* (Bleeker) | | | 287 | 0.93 | 39210.7 | | | 0.63 | | 24.4 | | | 14.5 [12] | | | |
| *Culter alburnus* Basilewsky | | | 241 | 0.78 | 153176.1 | | | 2.47 | | 29.6 | | | 32.6 [2] | | | |
| *Xenocypris davidi* Bleeker | | | 239 | 0.78 | 2141.0 | | | 0.03 | | 16.8 | | | 15.0 [1] | | | |
| *Silurus asotus* Linnaeus | | | 238 | 0.77 | 103746.0 | | | 1.67 | | 23.9 | | | 13.5 [13] | | | |
| *Ctenopharyngodon idellus* (Cuvier et Valenciennes) | | | 205 | 0.67 | 279631.2 | | | 4.50 | | 37.7 | | | 55.0 [1] | | | |
| *Aristichthys nobilis* (Richardson) | | | 199 | 0.65 | 611245.5 | | | 9.84 | | 50.6 | | | 82.5 [2] | | | |
| *Parabotia fasciata* Dabry | | | 190 | 0.62 | 736.5 | | | 0.01 | | 6.6 | | | 8.5 [14] | | | |
| *Rhodeus ocellatus kurumeus* | | | 180 | 0.59 | 837.3 | | | 0.01 | | 5.3 | | | 2.6 [15] | | | |
| *Monopterus albus* Zuiew | | | 142 | 0.46 | 3579.1 | | | 0.06 | | 40.3 | | |  | | | |
| *Pseudolaubuca sinensis* Bleeker | | | 142 | 0.46 | 4072.8 | | | 0.07 | | 16.5 | | |  | | | |
| *Abbottina rovularis* Basilewsky | | | 140 | 0.46 | 503.6 | | | 0.01 | | 5.9 | | |  | | | |
| *Pelteobagrus eupogon* (Boulenger) | | | 139 | 0.45 | 2677.9 | | | 0.04 | | 11.1 | | |  | | | |
| *Leiocassis longirostris* Gunther | | | 133 | 0.43 | 103045.0 | | | 1.66 | | 35.1 | | |  | | | |
| *Pseudorasbora parva* | | | 86 | 0.28 | 257.9 | | | <0.01 | | 5.4 | | |  | | | |
| *Sarcocheilichthys nigripinnis* | | | 82 | 0.27 | 2005.7 | | | 0.03 | | 10.4 | | |  | | | |
| *Misgurnus anguillicaudatus* (Cantor) | | | 80 | 0.26 | 1574.3 | | | 0.03 | | 11.9 | | |  | | | |
| *Channa argus* (Cantor) | | | 80 | 0.26 | 14230.9 | | | 0.23 | | 20.3 | | |  | | | |
| *Culter mongolicus mongolicus* (Basilewsky) | | | 79 | 0.26 | 32651.2 | | | 0.53 | | 25.6 | | |  | | | |
| *Hemibarbuslabeo* (Pallas) | | | 64 | 0.21 | 7036.7 | | | 0.11 | | 18.1 | | |  | | | |
| *Hemisalanx brachyrostralis*(Fang） | | | 61 | 0.20 | 73.6 | | | <0.01 | | 7.3 | | |  | | | |
| *Hemibarbus maculatus* Bleeker | | | 60 | 0.20 | 5230.9 | | | 0.08 | | 15.7 | | |  | | | |
| *Sinibotia superciliaris* (Günther) | | | 60 | 0.20 | 188.9 | | | <0.01 | | 8.6 | | |  | | | |
| *Leptobotia elongata* (Bleeker) | | | 59 | 0.19 | 15383.6 | | | 0.25 | | 26.2 | | |  | | | |
| *Silurus meridionalis* Chen | | | 54 | 0.18 | 82963.7 | | | 1.34 | | 50.1 | | |  | | | |
| *Pseudobagrus truncates* (Regan) | | | 53 | 0.17 | 1805.4 | | | 0.03 | | 14.6 | | |  | | | |
| *Erythroculter oxycephaloides* (Bleeker) | | | 51 | 0.17 | 8053.7 | | | 0.13 | | 17.7 | | |  | | | |
| *Megalobrama amblycephala* Yih | | | 51 | 0.17 | 38648.1 | | | 0.62 | | 28.8 | | |  | | | |
| *Saurogobio dumerili*(Bleeker) | | | 48 | 0.16 | 2237.1 | | | 0.04 | | 15.6 | | |  | | | |
| *Acheilognathus chankaensis* | | | 44 | 0.14 | 187.1 | | | <0.01 | | 5.0 | | |  | | | |
| *Rhodeus sinensis Günther* | | | 39 | 0.13 | 463.2 | | | 0.01 | | 13.1 | | |  | | | |
| *Erythroculter dabryi* (Bleeker) | | | 37 | 0.12 | 495.0 | | | 0.01 | | 10.4 | | |  | | | |
| *Mugilogobius myxodermus* (Herre) | | | 32 | 0.10 | 33.5 | | | <0.01 | | 3.9 | | |  | | | |
| *Hyporhamphus intermedius* (Cantor) | | | 31 | 0.10 | 410.6 | | | 0.01 | | 12.0 | | |  | | | |
| *Rhinogobius giurinus* (Rutter） | | | 29 | 0.09 | 85.7 | | | <0.01 | | 5.2 | | |  | | | |
| *Sarcocheilichthys sinensis* (Bleeker) | | | 28 | 0.09 | 346.6 | | | 0.01 | | 7.4 | | |  | | | |
| *Odontobutis obscura* (Temminck & Schlegel) | | | 27 | 0.09 | 590.3 | | | 0.01 | | 9.4 | | |  | | | |
| *Siniperca kneri* Garman | | | 24 | 0.08 | 1642.0 | | | 0.03 | | 14.0 | | |  | | | |
| *Culter oxycephaloides* Kreyenberg et Pappenheim | | | 24 | 0.08 | 4645.9 | | | 0.07 | | 24.7 | | |  | | | |
| *Rhodeus lighti* Wu | | | 21 | 0.07 | 23.4 | | | <0.01 | | 3.3 | | |  | | | |
| *Mastacembelus aculeatus* Basil | | | 19 | 0.06 | 225.6 | | | <0.01 | | 15.6 | | |  | | | |
| *Culter dabryi* | | | 19 | 0.06 | 550.6 | | | 0.01 | | 12.1 | | |  | | | |
| *Siniperca roulei* Wu | | | 18 | 0.06 | 1116.3 | | | 0.02 | | 15.2 | | |  | | | |
| *Saurogobio gymnocheilus* Lo Yao & Chen | | | 17 | 0.06 | 611.9 | | | 0.01 | | 14.7 | | |  | | | |
| *Mylopharyngodon pieces* Richardson | | | 17 | 0.06 | 89139.5 | | | 1.44 | | 48.5 | | |  | | | |
| *Macropodus chinensis* (Bloch) | | | 17 | 0.06 | 31.4 | | | <0.01 | | 3.7 | | |  | | | |
| *Coreius guichenoti* (Sauvage et Dabry) | | | 16 | 0.05 | 6609.6 | | | 0.11 | | 29.4 | | |  | | | |
| *Megalobrama terminalis* (Rich) | | | 13 | 0.04 | 4104.2 | | | 0.07 | | 20.8 | | |  | | | |
| *Leptobotia taeniaps* (Sauvage) | | | 13 | 0.04 | 161.3 | | | <0.01 | | 9.3 | | |  | | | |
| *Tinca tinca* (Linnaeus) | | | 12 | 0.04 | 3489.6 | | | 0.06 | | 22.7 | | |  | | | |
| *Leptobotia rubrilabris* (Dabry de Thiersant) | | | 12 | 0.04 | 86.5 | | | <0.01 | | 6.6 | | |  | | | |
| Pseudobagrus brevicaudatus (H. W. Wu) | | | 11 | 0.04 | 174.7 | | | <0.01 | | 10.3 | | |  | | | |
| *Myxocy prinus asiaticus* (Bleeker) | | | 11 | 0.04 | 7891.4 | | | 0.13 | | 24.3 | | |  | | | |
| *Pseudolaubuca engraulis* (Nichols) | | | 10 | 0.03 | 325.0 | | | 0.01 | | 13.7 | | |  | | | |
| *Ancherythroculter nigrocauda* Yih et Wu | | | 10 | 0.03 | 259.8 | | | <0.01 | | 12.4 | | |  | | | |
| *Lepturichthys fmbriata* (Gunther) | | | 10 | 0.03 | 60.5 | | | <0.01 | | 9.6 | | |  | | | |
| *Platysmacheilus exiguus* (Lin) | | | 10 | 0.03 | 367.0 | | | 0.01 | | 14.6 | | |  | | | |
| *Leiocassis longirostris* Gunther | | | 9 | 0.03 | 566.0 | | | 0.01 | | 18.4 | | |  | | | |
| *Elopichthys bambusa* (Rich) | | | 6 | 0.02 | 44226.2 | | | 0.71 | | 41.9 | | |  | | | |
| *Paramisgurnus dabryanus* Sauvage | | | 5 | 0.02 | 91.1 | | | <0.01 | | 11.7 | | |  | | | |
| *Abbottina brevirostris* | | | 5 | 0.02 | 8.5 | | | <0.01 | | 4.5 | | |  | | | |
| *Jinshaia sinensis* (Sauvage et Dabry) | | | 5 | 0.02 | 54.7 | | | <0.01 | | 9.8 | | |  | | | |
| *Culter molitorella* (Cuvier et Valenciennes) | | | 4 | 0.01 | 363.1 | | | 0.01 | | 18.1 | | |  | | | |
| *Gobiobotia flifer* (Garman) | | | 4 | 0.01 | 43.0 | | | <0.01 | | 9.3 | | |  | | | |
| *Pseudobagrus emarginatus* | | | 3 | 0.01 | 135.8 | | | <0.01 | | 13.6 | | |  | | | |
| *Cobitis taenia*Linnaeus | | | 3 | 0.01 | 110.9 | | | <0.01 | | 15.7 | | |  | | | |
| *Rhinogobio ventralis* Sauvage et Dabry | | | 3 | 0.01 | 377.4 | | | 0.01 | | 18.3 | | |  | | | |
| *Cobitis sinensis* (Sauvage et Dabry de Thiersant） | | | 3 | 0.01 | 4.0 | | | <0.01 | | 5.5 | | |  | | | |
| *Acipenser dabryanus* Dumeril | | | 2 | 0.01 | 958.5 | | | 0.02 | | 41.8 | | |  | | | |
| *Platysmacheilus nudiventris* Lo，Yao et Chen | | | 2 | 0.01 | 97.0 | | | <0.01 | | 16.1 | | |  | | | |
| *Opsariichthys bidens* Gunther | | | 2 | 0.01 | 37.2 | | | <0.01 | | 10.1 | | |  | | | |
| Acheilognathus gracilis Nichols | | | 2 | 0.01 | 4.9 | | | <0.01 | | 4.8 | | |  | | | |
| *Pseudobagrus tenuis*(Günther) | | | 2 | 0.01 | 19.8 | | | <0.01 | | 9.5 | | |  | | | |
| *Spinibarbus sinensis* (Bleeker) | | | 2 | 0.01 | 468.7 | | | 0.01 | | 21.6 | | |  | | | |
| *Parabotia fasciata Dabry* | | | 2 | 0.01 | 46.0 | | | <0.01 | | 13.3 | | |  | | | |
| *Liobagrus marginatus* (Günther) | | | 1 | <0.01 | 29.8 | | | <0.01 | | 15.3 | | |  | | | |
| *Acheilognathus macropterus* (Bleeker) | | | 1 | <0.01 | 8.4 | | | <0.01 | | 7.2 | | |  | | | |
| *Ochetobibus elongatus* (Kner) | | | 1 | <0.01 | 447.0 | | | 0.01 | | 38.3 | | |  | | | |
| *Distoechodon hupeinensis* Yih | | | 1 | <0.01 | 83.8 | | | <0.01 | | 16.7 | | |  | | | |
| *Sarcocheilichthys kiangsiensis nichols* | | | 1 | <0.01 | 12.0 | | | <0.01 | | 8.0 | | |  | | | |
| *Anguilla japonica*Temminck et Schlegel | | | 1 | <0.01 | 19.6 | | | <0.01 | | 24.5 | | |  | | | |
| *Toxabramis swinhonis* (Günther) | | | 1 | <0.01 | 44.0 | | | <0.01 | | 14.7 | | |  | | | |
| *Parabotia bimaculata* Chen | | | 1 | <0.01 | 4.5 | | | <0.01 | | 8.1 | | |  | | | |
| *Parabotia banarescui* (Nalbant) | | | 1 | <0.01 | 8.0 | | | <0.01 | | 8.5 | | |  | | | |
| *Hypseleotris swinhonis* (Günther) | | | 1 | <0.01 | 0.8 | | | <0.01 | | 3.3 | | |  | | | |
| *Distoechodon tumirostris* Peters | | | 1 | <0.01 | 162.1 | | | <0.01 | | 20.6 | | |  | | | |
| Total | | | 30761 | 100 | 6210956.7 | | | 100 | |  | | |  | | | |
|  |  | | | |  |  | |  | |  |  | |  |  |  |  |

Number (N), percentage number (% N), weight (in grams, W), percent weight (% W), and mean body length (mm, mean BL). *means that this species is peculiar to the middle reach of the Yangtze River.

## References

- - - 1. Zeng XC. Fishery Resources of the Yangtze River Basin. Beijing: Maine Press; 1990.
      2. Ichthyologic Department of Hubei Province. The Fishes of Yangtze River. Beijing: Science Press; 1976 (**in Chinese**).
      3. Duan ZH, Sun JY. Studies on the reproductive biology of *Pelteobagrus vachelli* (Richardson). Acta Hydrobiol Sinica. 1999;23:610-6 (**in Chinese with English abstract**).
      4. Xie ZY. Investigations of the biology of *Hemiculter Leuciscules* in Fenhe reservorir. J Shangdong Coll Oceanol. 1986;16:54-69 (**in Chinese with English abstract**).
      5. Wang DS, Tian HJ, Pu DY. Biological studies on *Leiocassis Crassilabris* Gunther: Bagride. J Southwest China Normal U (Nat Sci). 1995; 20:59-65 (**in Chinese with English abstract**).
      6. Wang HS. A Study of the Age，Growth and Mortality of *Squalidus argentatus* in Tian-e-zhou Oxbow of Yangtze River. J Hydroecol. 2013; 34:7-13 (**in Chinese with English abstract**).
      7. H SH. Preliminary studies on age, growth and reproduction of Yellow Catfish *Pseudobagrus nitidus* in Nanchang Section of Ganjiang River. MS Thesis, College of Fishery, Huazhong Agriculture University, Wuhan, China. 2010 (**in Chinese**).
      8. Shi BN. Biological studies on *Rhinogobio typus* Bleeker. J Southwest China Normal U. 1980;2:111-5 (**in Chinese**).
      9. Xu DP, Zhang MY, Zhou YF, Lai WT, Shi WG. Growth characteristics and morphological features of *Pseudobrama simoni* in Changshu section of Yangtze River. J Dalian Ocean U. 2014;29:397-402 (**in Chinese with English abstract**).
      10. Yu WJ. Age, growth, mortality and reproduction of *Hemiculter bleekeri* in He-Wang-Miao Oxbow. MS Thesis, College of Fishery, Huazhong Agriculture University, Wuhan, China. 2017 (**in Chinese**).
      11. Xiao TY, Zhang HY, Wang XQ, Xiao KY, Dai ZY. Biological Characteristics of *Pelteobagrus fulvidraco* in Dongting Lake. Chinese J Zool. 2003;38:83-8 (**in Chinese with English abstract**).
      12. Wang DS, Luo QS. Aspects of the reproductive biology of the bagridae catfish *Mystus macropterus* in the Jia Ling River. J Fish China. 1992;16:50-59 (**in Chinese with English abstract**).
      13. Wen HS, Mao YZ. The exploitation of resources and farming of wild catfish (*Silurus asotus* Linnaeus). Modern fish inform. 2003;18:6-9 (**in Chinese**).
      14. Yang MS. The age and growth characteristics of *Parabotia fasciata* in Huan River. J Xiaogan U. 2009;29:17-9 (**in Chinese with English abstract**).
      15. Zhang TL, Li ZJ, Cui YB. Age, growth and reproduction of *Rhodeus ocellatus* in the Niushan Lake, Hubei Province. J Lake Sci. 2002;14:267-72 (**in Chinese with English abstract**).
